# Frequent Mutations of VHL Gene and the Clinical Phenotypes in the Largest Chinese Cohort With Von Hippel–Lindau Disease

**DOI:** 10.3389/fgene.2019.00867

**Published:** 2019-09-18

**Authors:** Baoan Hong, Kaifang Ma, Jingcheng Zhou, Jiufeng Zhang, Jiangyi Wang, Shengjie Liu, Zhongyuan Zhang, Lin Cai, Ning Zhang, Kan Gong

**Affiliations:** ^1^Department of Urology, Peking University First Hospital, Beijing, China; ^2^Hereditary Kidney Cancer Research Center, Peking University First Hospital, Beijing, China; ^3^Institute of Urology, Peking University, Beijing, China; ^4^National Urological Cancer Center, Beijing, China; ^5^Department of Urology, Beijing Cancer Hospital, Beijing, China; ^6^Beijing Institute for Cancer Research, Beijing, China

**Keywords:** von Hippel–Lindau disease, VHL mutation, genotype–phenotype correlation, onset age, survival

## Abstract

Von Hippel–Lindau (VHL) disease is a rare autosomal-dominant inherited tumor syndrome. We aimed to analyze the correlations between frequent *VHL* mutations and phenotypes in Chinese VHL families. We screened 540 patients from 187 unrelated Chinese VHL families for 19 frequent VHL mutations. The penetrance and mean age at onset for VHL-associated susceptible organs were calculated and compared. The overall survival of VHL patients was described with Kaplan–Meier curves. Among the 19 frequent germline mutations, there were four hotspot mutation sites (194, 481, 499, and 500). Missense mutations were the most common types of mutations (70.0%) followed by nonsense mutations (20.0%) and splicing mutations (10.0%). Due to the diversity of these mutations, the penetrance for each organ and the age at onset are distinct. Even in cases of similar mutations, variance in the penetrance and age at onset was observed. The mean age at death for the patients in this cohort was 42.4 ± 13.5 years, and variability was observed in the Kaplan–Meier curves. We present a precise summary of the phenotypes for the frequent *VHL* mutations in the largest Chinese VHL cohort, which provides valuable strategies for genetic counseling and clinical surveillance of VHL individuals.

## Introduction

Von Hippel–Lindau (VHL) disease (OMIM no. 193300) is an autosomal-dominant familial neoplastic condition that is caused by germline mutations in the *VHL* gene located on chromosome 3p25-26. This gene comprises three exons: exon 1 spans nucleotides 1–340 (codons 1–113), exon 2 spans nucleotides 341–463 (codons 114–154), and exon 3 spans nucleotides 464–642 (codons 155–213) (Latif et al., 1993; Nielsen et al., 2016; Varshney et al., 2017). Patients with VHL syndrome inherit a single mutant *VHL* allele from a parent and develop the disease when the second wild-type copy is deactivated or lost. Incidence of the *VHL* mutation is approximately 1 in 36,000 live births, and it is greater than 90% penetrant by age 65 years (Kim et al., 2010; Gossage et al., 2015). Common VHL-associated clinical manifestations include central nervous system hemangioblastoma (CHB), renal cell carcinoma or renal cyst (RCC), retinal angioma (RA), pancreatic tumor or cyst (PCT), pheochromocytoma and paragangliomas (PHEO), endolymphatic sac tumor, and epididymis or broad ligament cystadenoma (**Supplementary Table 1**; Lonser et al., 2003; Lonser et al., 2004; Butman et al., 2008; Chou et al., 2013; Launbjerg et al., 2017). Von Hippel–Lindau disease predisposes the affected individuals to the development of lesions in multiple systems with CHBs (25%–51%) and RCCs (13%–47%) as the major causes of mortality (Grubb et al., 2005; Wilding et al., 2012; Lonser et al., 2014). Individuals with a family history of VHL will be clinically diagnosed when he/she presents with VHL-associated tumors that include CHB, RA, or RCC (Chittiboina and Lonser, 2015). For patients who do not have a family history of VHL, two or more CHBs or RAs or one hemangioblastoma and a visceral tumor are required for a clinical diagnosis (Nielsen et al., 2016). Typically, genetic testing is the standard method to diagnose VHL disease. The detection of *VHL* mutations not only contributes to an early and precise diagnosis of at-risk individuals but also helps to elucidate the genotype–phenotype correlations within a given population.

A series of studies have reported genotype–phenotype correlations in VHL diseases from different research perspectives or within different ethnic backgrounds (Yoshida et al., 2000; Patocs et al., 2008; Gomy et al., 2010; Nordstrom-O’Brien et al., 2010; Vikkath et al., 2015). For example, a retrospective study that included 63 VHL patients from two large VHL kindreds (family 1: Y112H mutation and family 2: Y98H mutation) with pheochromocytoma/paraganglioma found that pheochromocytoma expressivity differed by genotype (Nielsen et al., 2011). Ong et al. (2007) evaluated the genotype–phenotype correlations in 573 VHL patients and confirmed that pheochromocytoma was linked to *VHL* missense mutations. Additionally, the age at onset for VHL syndrome was significantly earlier (*P* = 0.001) and the age-related risks of RA and RCC were higher (*P* = 0.022 and *P* = 0.0008, respectively) for individuals with nonsense or frameshift mutations compared to those with deletions. Importantly, the results of these studies provided valuable strategies for genetic counseling and clinical prophylactic surveillance for VHL family members.

Due to the rarity of VHL disease, studies on the correlations between the frequent mutations of the *VHL* gene and clinical phenotypes are relatively scarce, with the majority being case reports or studies involving a limited number of VHL patients or families. In clinical practice, there is an urgent need to identify the clinical symptoms and survival statistics for VHL patients based on their specific types of mutations. Therefore, an improved and precise understanding of specific genotype–phenotype correlations in VHL disease is essential for targeted monitoring and counseling. In this study, we screened for frequent mutations in the *VHL* gene across 187 unrelated Chinese VHL families and analyzed the genotype–phenotype correlations between frequent *VHL* mutations and clinical manifestations. This study improves our understanding of how frequent mutations of the *VHL* gene affect the age at onset for each susceptible organ and their impact on prognosis in a Chinese population and provides a more accurate resource for genetic counseling and the monitoring of VHL patients.

## Materials and Methods

### Patient Selection

By May 31, 2018, 540 patients from 187 unrelated families had been diagnosed with VHL disease at the Peking University First Hospital, and all were screened in the present study. Any mutation site that appeared in two or more families was included in this study. Individuals who carried a *VHL* germline mutation or who met the clinical criteria were diagnosed with VHL disease (Wu et al., 2012). The evidence assessment for VHL disease includes a medical history, a physical examination by multidisciplinary teams, laboratory tests, and medical imaging of the abdomen, pelvis, brain, and spine (ultrasound, computed tomography, or magnetic resonance imaging). However, at least one patient from a family confirmed the presence of a *VHL* mutation through genetic testing.

Clinical characteristics including date of birth, age at death, mutation type, clinical symptoms, and the age at onset were collected from family members or through medical records. The age at onset was defined as the age at which VHL-related symptoms or signs first appear. A follow-up was performed on all patients and asymptomatic carriers in the VHL families to determine the age at onset for six major VHL-related lesions: CHB, RA, RCC, PCT, PHEO, and GS (genital system including the epididymis or broad ligament). Follow-ups were carried out until May 31, 2018, or until patient death following the first presentation of the VHL-related clinical symptoms or a positive genetic diagnosis of VHL disease. The life span of the patient from birth until death or until the end of the follow-up period was used in the survival analysis.

### Genetic Analysis

Genetic testing was performed on at least one member from each family to confirm the diagnosis of VHL disease. Genomic DNA was extracted from peripheral blood samples that were obtained from members of the suspected families. Individuals who refused the genetic test or who died were excluded. The three exons and the flanking intronic sequences of the *VHL* gene were amplified by polymerase chain reaction, and direct sequencing was used to detect missense mutations, splicing mutations, and small indels. Large deletions and duplications were detected by multiplex ligation-dependent probe amplification (MLPA, P016-C2 kit, Amsterdam, the Netherlands) and verified by real-time quantitative polymerase chain reaction. The primers and reaction conditions used for the amplification were as previously described (Peng et al., 2017; Wang et al., 2018). The spectrum of *VHL* mutations was screened for frequent mutations and further analyzed to determine their correlations with phenotypes.

### Statistical Analysis

For each type of mutation, the mean age at onset of VHL-associated susceptible organs (CHB, RA, RCC, PCT, PHEO, and GS) and the mean age at death were calculated as the mean ± standard deviation. Statistical significance was determined by the Student *t* test or the LSD multiple-comparisons *t* test. The overall survival of VHL patients was described with a Kaplan–Meier curve. Statistical analysis was performed using SPSS 20.0 software (IBM-SPSS, Inc., Chicago, IL, USA), and *P* < 0.05 was considered to be statistically significant.

## Results

### Clinical Characteristics of Chinese VHL Patients and the Distribution of Frequent Germline Mutations

**Figure 1:**
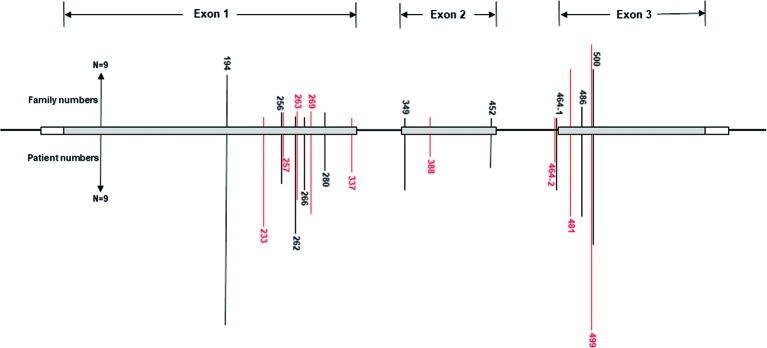
Distribution of the frequent germline mutation sites in *VHL* gene for 258 patients from 80 unrelated Chinese families with VHL disease. Of the 19 frequent germline mutations, 10 were located in exon 1, 3 were located in exon 2, 4 were located in exon 3, and 2 were located in intron 2. Notably, there were four hotspot mutation sites (194, 481, 499, and 500). The bars on the upper part represent mutational family numbers, and those on the lower part represent mutational patient numbers.

A total of 540 patients from 187 unrelated Chinese VHL families were included in our database, and 126 different types of *VHL* mutations were identified. Insertions, small deletions, and large deletions were detected in 61 families [61/187 (32.6%)]. Point mutations resulting in missense, nonsense, or splicing mutations were detected in 126 families [126/187 (67.4%)]. The distribution of germline point mutations was further analyzed and identified 19 germline mutation sites that appeared in two or more families. Of these 19 frequent germline mutations, 10 were located in exon 1, 3 were located in exon 2, 4 were located in exon 3, and 2 were located in intron 2 (**Figure 1**). The clinical characteristics and mutation frequencies for each of the relevant germline mutations in the 258 patients from 80 unrelated Chinese VHL families are shown in **Table 1**. The mean age of the 258 patients was 39.4 ± 15.5 years with a range of 3 to 74 years. Additionally, the mean age for each mutation group is listed in **Table 1**. As would be expected due to the consequences of frequent mutations, missense mutations were the most common mutation types in these families (70.0%) followed by nonsense mutations (20.0%) and splicing mutations (10.0%). Notably, there were four mutation hotspot sites at 194, 481, 499, and 500 that were separately distributed across 9, 10, 14, and 10 unrelated Chinese VHL families, respectively.

**Table 1:** Clinical characteristics and mutation frequency of relevant frequent germline mutations of 258 patients from 80 unrelated Chinese VHL families.

Mutation sites	Mean age	Exon	Mutation type	FAMILY NUMBERS		Patient numbers	
				n	%	n	%
194	39.6 ± 16.3	1	Missense	9	4.81	31	5.74
233	34.6 ± 19.5	1	Missense	2	1.07	19	3.52
256	35.0 ± 15.8	1	Missense	3	1.60	9	1.67
257	30.3 ± 8.0	1	Missense	3	1.60	4	0.74
262	45.1 ± 10.1	1	Missense	2	1.07	21	3.89
263	33.8 ± 17.6	1	Missense Nonsense	3	1.60	13	2.41
266	36.6 ± 15.3	1	Missense	2	1.07	11	2.04
269	36.9 ± 15.6	1	Missense	3	1.60	15	2.78
280	42.0 ± 12.6	1	Nonsense	3	1.60	6	1.11
337	47.0 ± 18.4	1	Nonsense	2	1.07	6	1.11
349	44.2 ± 15.5	2	Missense	2	1.07	11	2.04
388	41.8 ± 10.1	2	Missense	2	1.07	4	0.74
452	39.0 ± 13.1	2	Missense	2	1.07	7	1.30
464-1	28.8 ± 19.6	Intron 2	Splicing	2	1.07	11	2.04
464-2	42.6 ± 17.9	Intron 2	Splicing	2	1.07	5	0.93
481	39.2 ± 10.4	3	Nonsense	10	5.35	15	2.78
486	38.3 ± 13.9	3	Missense	4	2.14	15	2.78
499	38.2 ± 17.4	3	Missense	14	7.49	31	5.74
500	47.8 ± 12.7	3	Missense	10	5.35	24	4.44

### Correlations Between Frequent VHL Germline Mutations and Clinical Phenotypes in Chinese VHL Patients

**Figure 2:**
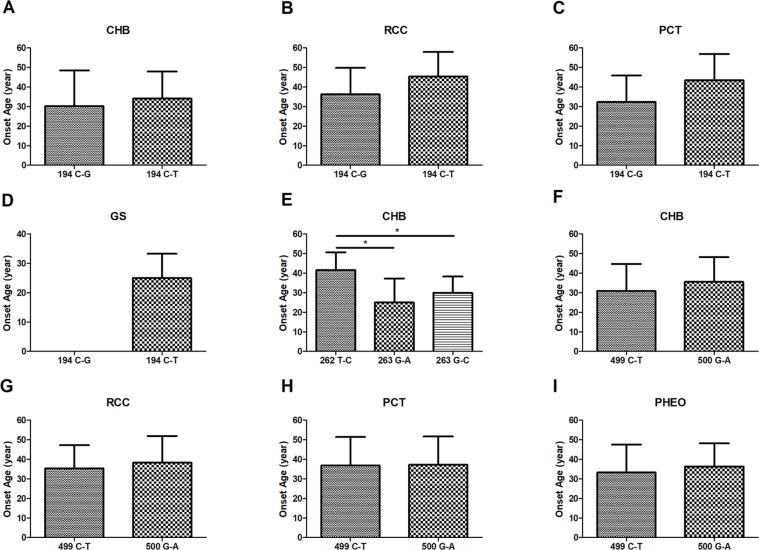
The mean age at onset for the common VHL lesions in patients with different germline mutations. The mean age at onset for the common VHL lesions in the c.194C > T p.Ser65Leu mutational subgroup and the c.194C > G p.Ser65Trp mutational subgroup **(A**–**D)**. Comparison of the mean age at onset for CHB among c.262T > C p.Trp88Arg, c.263G > A p.Trp88Stop, and c.263G > C p.Trp88Ser mutations **(E)**. Differences in the mean age at onset for the four major VHL lesions between the c.499C > T p.Arg167Trp and c.500G > A p.Arg167Gln mutations **(F**–**I**, *P* > 0.05**)**. *P < 0.05.

In this study, we analyzed and compared the mean age at onset for six major VHL-related lesions (CHB, RA, RCC, PCT, PHEO, and GS) in 19 frequent germline mutations (**Table 2**). Due to diversity in the types of mutations, the penetrance for each organ and the age at onset are not the same. For CHB, both the c.481C > T p.Arg161stop (group 16) and c.486C > G p.Cys162Trp (group 17) mutations had a high penetrance of approximately 80.0% (12/15). The mean age at onset of CHB for the c.481C > T p.Arg161stop (group 16) mutation was 27.4 ± 9.4 years (range = 14–40 years), while for the c.486C > G p.Cys162Trp (group 17) mutation, it was 31.4 ± 10.0 years (range = 12–49 years). For RCC, the mean ages at onset for the c.269A > T p.Asn90Ile (group 8) and c.486C > G p.Cys162Trp (group 17) mutations were 41.5 ± 15.5 years and 41.8 ± 10.3 years, while the penetrance was 26.7% (4/15) and 53.3% (8/15), respectively. Variation in the penetrance and age at onset exists even when the types of mutations are similar. There were two types of missense mutations in group 1 located in the 194 mutation site, c.194C > T p.Ser65Leu and c.194C > G p.Ser65Trp, but the clinical phenotypes were different between these two mutational subgroups. Six major VHL-related lesions (CHB, RA, RCC, PCT, PHEO, and GS) were observed in the c.194C > T p.Ser65Leu mutational subgroup, while only three VHL lesions (CHB, RCC, and PCT) presented in the c.194C > G p.Ser65Trp mutational subgroup. The mean age at onset for the common VHL lesions in the c.194C > T p.Ser65Leu mutational subgroup was older than that of the c.194C > G p.Ser65Trp mutational subgroup (**Figures 2A–D**). Three mutations were related to codon 88 (Trp) in groups 5 and 6. Both c.262T > C p.Trp88Arg and c.263G > C p.Trp88Ser were missense mutations, while c.263G > A p.Trp88Stop resulted in a nonsense mutation. Additionally, the VHL lesions associated with these three mutations were not the same. A comparison was made between the mean age at onset for CHB in these three subgroups and found that the c.262T > C p.Trp88Arg mutational group was older than that of the c.263G > C p.Trp88Ser mutational subgroup (*P* = 0.0152) and the c.263G > A p.Trp88Stop mutational subgroup (*P* = 0.0232) (**Figure 2E**). However, the CHB-associated age at onset for the c.263G > A p.Trp88Stop mutational subgroup was younger than the c.263G > C p.Trp88Ser mutational subgroup, but the difference was not significant (*P* = 0.481) (**Figure 2E**). In groups 18 and 19, the c.499C > T p.Arg167Trp and c.500G > A p.Arg167Gln mutations were located in codon 167 (Arg). There were differences in the penetrance and age at onset for six major VHL-related lesions (CHB, RA, RCC, PCT, PHEO, and GS) between these two mutation types, but the difference was not statistically significant (*P* > 0.05) (**Figures 2F–I**).

**Table 2:** Frequent *VHL* germline mutations and related phenotypes in Chinese patients with VHL disease.

Group	NC	CHB		RA		RCC		PCT		PHEO		GS	
		Ratio (%)	OA (Mean ± SD)	Ratio (%)	OA (Mean ± SD)	Ratio (%)	OA (Mean ± SD)	Ratio (%)	OA (Mean ± SD)	Ratio (%)	OA (Mean ± SD)	Ratio (%)	OA (Mean ± SD)
1*	c.194C > T p.Ser65Leu	62.5 (15/24)	34.1 ± 13.8 (15–59)	8.3 (2/24)	(30, 53)	37.5 (9/24)	45.4 ± 12.5 (25–60)	45.8 (11/24)	43.5 ± 13.5 (25–65)	4.2 (1/24)	(34)	16.7 (4/24)	25.0 ± 8.3 (17–34)
	c.194C > G p.Ser65Trp	71.4 (5/7)	30.2 ± 18.3 (17–61)	—	—	57.1 (4/7)	36.3 ± 13.6 (24–48)	42.9 (3/7)	32.3 ± 13.6 (24–48)	—	—	—	—
2	c.233A > G p.Asn78Ser	21.1 (4/19)	41.8 ± 10.4 (30–51)	—	—	15.8 (3/19)	38.3 ± 2.5 (36–41)	36.8 (7/19)	27.4 ± 6.9 (20–40)	10.5 (2/19)	(28, 36)	—	—
3	c.256C > T p.Pro86Ser	33.3 (3/9)	34.0 ± 2.6 (31–36)	22.2 (2/9)	(11,12)	55.6 (5/9)	34.0 ± 4.1 (30–40)	33.3 (3/9)	33.3 ± 2.5 (31–36)	11.1 (1/9)	(31)	—	—
4	c.257C > T p.Pro86Leu	75.0 (3/4)	26.3 ± 8.0 (18–34)	50.0 (2/4)	(26, 32)	75.0 (3/4)	28.0 ± 9.2 (20–38)	75.0 (3/4)	27.0 ± 10.5 (17–38)	—	—	25.0 (1/4)	(26)
5	c.262T > C p.Trp88Arg	52.4 (11/21)	41.1 ± 9.1 (29–62)	—	—	38.1 (8/21)	37.4 ± 12.9 (23–65)	4.8 (1/21)	(30)	4.8 (1/21)	(50)	—	—
6*	c.263G > A p.Trp88Stop	75.0 (3/4)	25.0 ± 12.3 (11–34)	—	—	50.0 (2/4)	(28, 34)	50.0 (2/4)	(28, 38)	25.0 (1/4)	(39)	50.0 (2/4)	(11,32)
	c.263G > C p.Trp88Ser	77.8 (7/9)	29.9 ± 8.4 (20–44)	22.2 (2/9)	(25, 35)	11.1 (1/9)	(30)	22.2 (2/9)	(30, 31)	—	—	11.1 (1/9)	(24)
7	c.266T > C p.Leu89Pro	54.5 (6/11)	27.7 ± 7.5 (20–40)	45.5 (5/11)	29.6 ± 13.1 (15–42)	54.5 (6/11)	39.5 ± 9.3 (24–52)	36.4 (4/11)	25.8 ± 2.2 (24–29)	9.1 (1/11)	(45)	—	—
8	c.269A > T p.Asn90Ile	66.7 (10/15)	29.9 ± 13.4 (16–63)	13.3 (2/15)	(23, 30)	26.7 (4/15)	41.5 ± 15.5 (26–63)	33.3 (5/15)	41.4 ± 17.0 (17–63)	6.7 (1/15)	(38)	—	—
9	c.280G > T p.Glu94Stop	66.7 (4/6)	34.5 ± 13.5 (23–54)	33.3 (2/6)	(17, 29)	50.0 (3/6)	35.0 ± 15.9 (23–53)	33.3 (2/6)	(29, 53)	16.7 (1/6)	(35)	33.3 (2/6)	(14, 20)
10	c.337C > T p.Arg113Stop	50.0 (3/6)	40.0 ± 22.5 (28–66)	50.0 (3/6)	23.0 ± 7.2 (17–31)	50.0 (3/6)	35.3 ± 11.3 (26–48)	33.3 (2/6)	(26, 27)	—	—	16.7 (1/6)	(18)
11	c. 349T > G p.Trp117Gly	72.7 (8/11)	30.0 ± 14.6 (12–47)	45.4 (5/11)	23.8 ± 18.2 (7–44)	54.5 (6/11)	45.8 ± 8.1 (37–61)	72.7 (8/11)	43.4 ± 14.3 (21–59)	9.1 (1/11)	(18)	9.1 (1/11)	(44)
12	c.388G > C p.Val130Leu	50.0 (2/4)	(30, 30)	—	—	25.0 (1/4)	(31)	25.0 (1/4)	(31)	25.0 (1/4)	(30)	—	—
13	c.452T > G p.Ile151Ser	71.4 (5/7)	34.2 ± 18.1 (14–60)	14.3 (1/7)	(26)	42.9 (3/7)	36.7 ± 11.7 (28–50)	28.6 (2/7)	(32, 38)	—	—	—	—
14	Intron 2 ^#^ c.464–1 G > C	54.5 (6/11)	33.0 ± 18.4 (16–66)	—	—	36.4 (4/11)	42.3 ± 16.5 (28–66)	36.4 (4/11)	42.3 ± 16.5 (28–66)	—	—	9.1 (1/11)	(10)
15	Intron 2 ^#^ c.464-2 A > G	40.0 (2/5)	(24, 57)	20.0 (1/5)	(57)	60.0 (3/5)	40.3 ± 16.8 (21–51)	60.0 (3/5)	40.3 ± 16.8 (21–51)	20.0 (1/5)	(58)	—	—
16	c.481C > T p.Arg161Stop	80.0 (12/15)	27.4 ± 9.4 (14–40)	6.7 (1/15)	(28)	66.7 (10/15)	36.6 ± 8.2 (23–47)	60.0 (9/15)	32.3 ± 9.7 (14–46)	6.7 (1/15)	(41)	13.3 (2/15)	(30, 36)
17	c.486C > G p.Cys162Trp	80.0 (12/15)	31.4 ± 10.0 (12–49)	26.7 (4/15)	36.5 ± 16.3 (17–54)	53.3 (8/15)	41.8 ± 10.3 (29–56)	40.0 (6/15)	33.0 ± 12.9 (17–56)	6.7 (1/15)	(29)	6.7 (1/15)	(23)
18	c.499C > T p.Arg167Trp	35.5 (11/31)	30.9 ± 13.8 (16–65)	12.9 (4/31)	23.5 ± 8.7 (13–34)	22.6 (7/31)	35.4 ± 11.8 (18–54)	29.0 (9/31)	36.9 ± 14.5 (19–67)	32.3 (10/31)	33.3 ± 14.3 (12–58)	3.2 (1/31)	(31)
19	c.500G > A p.Arg167Gln	62.5 (15/24)	35.6 ± 12.6 (15–52)	16.7 (4/24)	23.0 ± 6.8 (14–30)	41.7 (10/24)	38.3 ± 13.6 (22–54)	20.8 (5/24)	37.2 ± 14.5 (22–53)	37.5 (9/24)	36.3 ± 11.9 (14–52)	—	—

*There are two mutational types in this site. ^#^Splicing mutation.

—, no patient with this phenotype; NC, nucleotide change and consequence; OA, onset age (year); CHB, central nervous system hemangioblastoma; RA, retinal angioma; RCC, renal cell carcinoma or cyst; PCT, pancreatic tumor or cyst; PHEO, pheochromocytoma; GS, genital system (epididymis or broad ligament).

### Frequent VHL Germline Mutations and Survival

**Figure 3:**
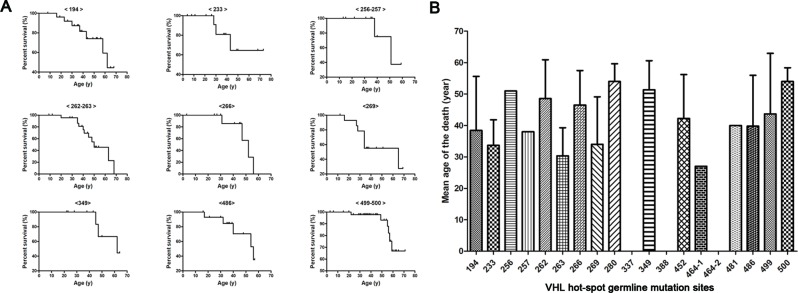
Von Hippel–Lindau frequent germline mutations and survival. Kaplan–Meier curves were used to describe the survival of patients with different *VHL* mutations **(A)**. Variability was observed in the mean age at death for the different frequent VHL germline mutations, but no death was observed in groups with mutation sites 337, 388 and 464-2 **(B)**.

Kaplan–Meier curves were used to describe the survival of patients with different *VHL* mutations, and the results are presented in **Figure 3A**. This analysis identified a variety of Kaplan–Meier curves for different frequent *VHL* germline mutations. The mutation sites 256 and 257, 262 and 263, and 499 and 500 were located in codons 86, 88, and 167, respectively, and grouped together for the analysis. Of the 258 patients from the 80 unrelated Chinese VHL families, 59 died of VHL-related diseases, such as CHB [71.2% (42/59)], RCC [25.4% (15/59)], and PCT-related complications [3.4% (2/59)]. The mean age at death for this cohort was 42.4 ± 13.5 years (range = 16–68 years). Additionally, we summarized the mean age at death for each of the frequent *VHL* germline mutation groups, and no deaths were observed in the groups with mutation sites 337, 388, and 464-2 (**Figure 3B**). Differences in the risk of VHL-related death across the different frequent *VHL* germline mutations were not statistically significant.

## Discussion

A comprehensive understanding of the correlations between genotype and phenotype for hereditary diseases is critical to the clinical management and scientific analysis of their pathogenesis. Changes in specific genotypes can lead to alterations in protein expression patterns that result in their corresponding phenotypes. Elucidating these correlations may provide insight into the molecular pathogenesis of the individual manifestations of VHL syndrome. Screening for mutations in the *VHL* gene helps to clarify the diagnosis of asymptomatic first-degree relatives, thereby improving patient outcomes through early disease surveillance. To date, the studies on genotype–phenotype correlations have provided clinicians with tools that help predict the VHL disease processes in individual patients. Hence, it is important to increase the sample size and analyze the correlations between genotypes and phenotypes for different ethnic groups.

In this study, we analyzed the correlations between frequent mutations in the *VHL* gene and clinical phenotypes in the largest Chinese VHL cohort to date. In total, we screened 540 patients from 187 unrelated VHL families and identified 126 different VHL mutations. Furthermore, we identified 19 frequent mutations and four mutation hotspots and further investigated the genotype–phenotype correlations. Notably, patients or families with the VHL disease have a range of different phenotypes. A variety of factors may contribute to this diversity of phenotypes, including the type of *VHL* mutation, the site of the mutation, and ethnic background.

Different ethnic backgrounds are associated with diverse phenotypes. Several studies about Western and Japanese populations highlighted the differences in the spectrum of *VHL* germline mutations (Maher et al., 1996; Patel et al., 2000). The mutation hotspots of the *VHL* gene that are already known include Leu178, Cys162, Arg167, Asn78, Pro86, and Tyr98 and have a frequency of approximately 3% to 17% (Stebbins et al., 1999). However, the common mutations are varied across different ethnic groups. Hwang et al. (2014) reported that Glu70Lys was a high-frequency *VHL* germline mutation in the Korean population, with nine unrelated patients [16.4% (9/55)] who had the same amino-acid alteration at codon 70 (Glu70Lys) and exhibited VHL type 1 phenotypes. However, in our cohort, the high-frequency mutations included Ser65 (4.81%), Arg161 (5.35%), and Arg167 (12.84%). Thus, the spectrum of *VHL* mutations varies in countries that have different ethnic backgrounds. Patients from different ethnic backgrounds that have the same *VHL* germline mutation may also develop distinct phenotypes. For example, Yoshida et al. (2000) found four mutations (Arg113Stop, Gln132Stop, Leu158Val, and Cys162Tyr) in Japanese families with the VHL type 2 phenotype, whereas Crossey et al. (1994) reported that these mutations were associated with the VHL type 1 phenotype in Western populations. Similarly, c.500G > A p.Arg167Gln is a hotspot mutation in many populations. Studies in Western populations showed that the mutation of c.500G > A p.Arg167Gln was associated with RCC and renal cysts, indicating that this mutation was associated with the VHL type 1 phenotype (Hes et al., 2007; Ciotti et al., 2009). However, according to our database, we found that this mutation was also related to PHEO (37.5%, 9 of 24), which indicated that the phenotype of the c.500G > A p.Arg167Gln mutation also differs in different ethnic backgrounds.

Different mutations may produce remarkably diverse phenotypes. For example, in group 1, two missense mutations occurred in the 194 mutation site, c.194C > T p.Ser65Leu and c.194C > G p.Ser65Trp. Intriguingly, the six major VHL-related lesions (CHB, RA, RCC, PCT, PHEO, and GS) were observed in the c.194C > T p.Ser65Leu mutational subgroup, while only three VHL lesions (CHB, RCC, and PCT) presented in the c.194C > G p.Ser65Trp mutational subgroup. Therefore, it indicates that missense mutations of different nucleotides in the same codon have potential effects on the clinical manifestation. The much lower number of patients in c.194C > G p.Ser65Trp mutational subgroup may limit the observation of the six major VHL-related lesions. This finding needs to be corroborated in larger cohorts. Bradley et al. (1999) also reported on the phenotypes of two distinct missense mutations in the same codon of the *VHL* gene (c.334T > A p.Tyr112Asn and c.334T > C p.Tyr112His). Thirteen patients were found with the c.334T > A p.Tyr112Asn mutation, seven of whom had RCC, and one of these patients had a pheochromocytoma, which suggests that this type of mutation causes the VHL type 1 phenotype, as most of the patients presented with RCC. Conversely, the c.334T > C p.Tyr112His mutation was associated with the VHL type 2A phenotype, as every affected individual in two families (22 patients) had PHEO but did not have RCC. Thus, different amino-acid changes at the same position may have different effects on the stability of the VHL protein, resulting in distinct clinical phenotypes.

**Figure 4:**
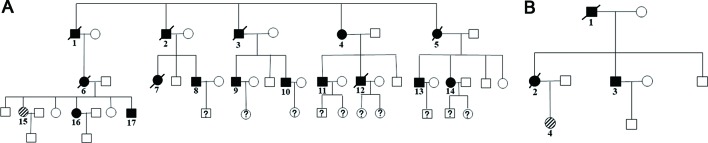
Two family pedigrees of group 5. □ and ○ indicate normal males and females, ▪ and • represent males and females with *VHL* mutation, and patient 15 in family 1 **(A)** and patient 4 in family 2 **(B)** were the probands.

Family members with the same mutation in *VHL* can also display different phenotypes. Mete et al. (2014) evaluated the clinical presentation of 49 family members from three generations of a Turkish family and identified the *VHL* p.A149S mutation. All of the patients were diagnosed with VHL syndrome type 2B, while nine patients were diagnosed with a pheochromocytoma, and one patient was diagnosed with a lumbar spinal hemangioblastoma and a pancreatic neuroendocrine tumor without pheochromocytoma. In our study, group 5 represented the c.262T > C p.Trp88Arg mutation that included 21 patients from 2 families (**Figure 4** and **Table 3**). Variability was observed in the mean age at onset for CHB and RCC, which were 41.1 ± 9.1 (range = 29–62) and 37.4 ± 12.9 (range = 23–65), respectively. Moreover, the penetrance of VHL lesions was also distinct. Taken together, this phenotypic variability suggests that other factors or molecular mechanisms may affect the phenotypes caused by specific mutations, such as environmental factors or telomere length (Ning et al., 2014; Wang et al., 2017).

**Table 3:** Characteristics of genotype and phenotype in group 5.

Family no.	Patient No.	Gender	Age	NC	Age at onset (y)					
					CHB	RA	RCC	PCT	PHEO	GS
1	1	Male	68*	c.262T > C p.Trp88Arg	—	—	65	—	—	—
	2	Male	58*		—	—	—	—	—	—
	3	Male	63*		62	—	—	—	—	—
	4	Female	66		37	—	—	—	—	—
	5	Female	48*		—	—	—	—	—	—
	6	Female	35*		35	—	—	—	—	—
	7	Female	41*		41	—	—	—	—	—
	8	Male	48		40	—	—	—	—	—
	9	Male	51		35	—	42	—	—	—
	10	Male	44		29	—	—	—	—	—
	11	Male	45		—	—	35	—	—	—
	12	Male	40*		—	—	40	—	—	—
	13	Male	39		39		37			
	14	Female	41		37		37			
	15 (Proband)	Female	32		—	—	30	30	—	—
	16	Female	28		—	—	27	—	—	—
	17	Male	23		—	—	—	—	—	—
2	1	Male	58*		48	—	—	—	—	—
	2	Female	45*			—	—	PC	—	—
	3	Male	52		50	—	—	—	50	—
	4 (Proband)	Female	31		—	—	—	—	—	—

*Patient was dead. NC, nucleotide change and consequence; PC, pancreatic cancer.

A classification of VHL diseases was proposed that was based on the patient’s preference for PHEO development. For example, the VHL type 1 phenotype has a low risk of PHEOs compared to the VHL type 2 phenotype, which is associated with PHEOs (Ong et al., 2007). The VHL type 2 phenotype is further classified into type 2A (hemangioblastoma and PHEO, but rarely RCC), type 2B (hemangioblastoma, PHEO, and RCC), and type 2C (PHEO only). However, during follow-up, the VHL disease shows characteristics of phenotypic variability, and new clinical manifestations may appear during the patient’s lifetime. Recently, Liu et al. (2018) reported genotype–phenotype correlations in VHL disease based on the alteration of a HIF-α binding site in the VHL protein. Lee et al. (2016) analyzed the genotype–phenotype correlations of VHL syndrome in Korean families and concluded that missense mutations in the Hypoxia-inducible factor-α (HIF-α) binding site elevate the age-specific risk for CHB. The studies cited above linked mutations in the *VHL* gene, protein binding sites, and phenotypic diversity to provide insights into the genotype–phenotype correlations based on amino-acid changes in the HIF-α binding site. In this study, we provide a precise summary of the penetrance and overall survival for each of the frequent mutations in the *VHL* gene within the largest Chinese VHL cohort. Our findings provide a more precise and individualized dataset that can be used in genetic counseling and research of the disease pathogenesis.

The current study had several limitations. Von Hippel–Lindau disease is rare, the size of this cohort is relatively small, and the follow-up durations are not sufficiently long, which may influence the correlation analysis between the frequent mutations in the *VHL* gene and the clinical phenotypes. Prospective, large-scale, and long-term follow-up studies are needed to further validate these results. Ultimately, elucidating the genotype–phenotype correlations of VHL disease will help to predict the risk of developing the diverse range of VHL-related phenotypes and their prognoses for individuals with VHL.

## Ethics Statement

The involvement of human participants in this study was based on the Declaration of Helsinki. The ethics of this study were reviewed and approved by the Institutional Ethics Committee of Peking University First Hospital. Informed consent to use clinical data was received from each patient or from their legal guardian.

## Author Contributions

Conceptualization: BH. Data curation: KM, JCZ, JFZ, JW, and SL. Formal analysis: BH. Funding acquisition: KG. Methodology: ZZ and LC. Project administration and supervision: KG and NZ. Original draft writing: BH.

## Funding

This work was supported by the National Natural Science Foundation of China (grant 81572506), the Special Health Development Research Project of Capital (grant 2016-2-4074), and the Fundamental Research Funds for the Central Universities (grant BMU2018JI002).

## Conflict of Interest Statement

The authors declare that the research was conducted in the absence of any commercial or financial relationships that could be construed as a potential conflict of interest.
