# Supplementary material for: Frequent Mutations of VHL Gene and the Clinical Phenotypes in the Largest Chinese Cohort With Von Hippel–Lindau Disease
Source: Front Genet. 2019 Sep 18;10:867. doi: 10.3389/fgene.2019.00867 (PMC6759728; doi:10.3389/fgene.2019.00867)
Supplement: Supplementary file 1 [file Table_1.docx]

**Supplementary Table 1.** Clinical manifestations of VHL patients

| **Organs** | **Clinical manifestations** |
| --- | --- |
| Central nervous system hemangioblastoma (CHB) | Cerebellum: ataxia, dysmetria, headache, vertigo, emesis Brainstem: hypaesthesia, ataxia, dysphagia, hyper-reflexia, headache Spinal cord: hypaesthesia, weakness, ataxia, hyper-reflexia |
| Retinal angioma (RA) | Blurred vision, part or total loss of vision (due to tractional retinal detachment ) |
| Renal cell carcinoma (RCC) | Advanced patient may present with haematuria, flank pain, abdominal mass |
| Pancreatic tumor or cyst (PCT) | Pancreatic cyst: generally asymptomatic Pancreatic neuroendocrine tumor: most are non-functional, few patient manifested with dyspepsia, backache, hyperglycemia |
| Pheochromocytoma and paragangliomas (PHEO) | Intermittent or sustained hypertension, tachycardia, headache, palpitation |
| Genital system (GS) | Epididymal cystadenoma: generally asymptomatic, few people with epididymal knob through palpation Broad ligament cystadenoma: generally asymptomatic |
